# RibM from *Streptomyces davawensis *is a riboflavin/roseoflavin transporter and may be useful for the optimization of riboflavin production strains

**DOI:** 10.1186/1472-6750-11-119

**Published:** 2011-12-02

**Authors:** Sabrina Hemberger, Danielle B Pedrolli, Jürgen Stolz, Christian Vogl, Martin Lehmann, Matthias Mack

**Affiliations:** 1 Institut für Technische Mikrobiologie, Hochschule Mannheim, 68163 Mannheim, Germany; 2 Zentralinstitut für Ernährungs- und Lebensmittelforschung (ZIEL), Lehrstuhl für Ernährungsphysiologie, Technische Universität München, Gregor-Mendel-Str. 2, 85350 Freising-Weihenstephan, Germany; 3 Biotechnology R&D, DSM Nutritional Products, P.O. Box 3255, CH-4002 Basel, Switzerland

## Abstract

**Background:**

The bacterium *Bacillus subtilis*, which is not a natural riboflavin overproducer, has been converted into an excellent production strain by classical mutagenesis and metabolic engineering. To our knowledge, the enhancement of riboflavin excretion from the cytoplasm of overproducing cells has not yet been considered as a target for (further) strain improvement. Here we evaluate the flavin transporter RibM from *Streptomyces davawensis *with respect to improvement of a riboflavin production strain.

**Results:**

The gene *ribM *from *S. davawensis*, coding for a putative facilitator of riboflavin uptake, was codon optimized (*ribM_opt_*) for expression in *B. subtilis*. The gene *ribM_opt _*was functionally introduced into *B. subtilis *using the isopropyl-β-thiogalactopyranoside (IPTG)-inducible expression plasmid pHT01: Northern-blot analysis of total RNA from IPTG treated recombinant *B. subtilis *cells revealed a *ribM_opt _*specific transcript. Western blot analysis showed that the his_6_-tagged heterologous gene product RibM was present in the cytoplasmic membrane. Expression of *ribM *in *Escherichia coli *increased [^14^C]riboflavin uptake, which was not affected by the protonophore carbonyl cyanide *m*-chlorophenylhydrazone (CCCP). Expression of *ribM_opt _*supported growth of a *B. subtilis *Δ*ribB*::Erm^r ^Δ*ribU*::Kan^r ^double mutant deficient in riboflavin synthesis (Δ*ribB*) and also deficient with respect to riboflavin uptake (Δ*ribU*). Expression of *ribM_opt _*increased roseoflavin (a toxic riboflavin analog produced by *S. davawensis*) sensitivity of a *B. subtilis *Δ*ribU*::Kan^r ^strain. Riboflavin synthesis by a model riboflavin *B. subtilis *production strain overproducing RibM was increased significantly depending on the amount of the inducer IPTG.

**Conclusions:**

The energy independent flavin facilitator RibM could in principle catalyze riboflavin export and thus may be useful to increase the riboflavin yield in a riboflavin production process using a recombinant RibM overproducing *B. subtilis *strain (or any other microorganism).

## Background

Riboflavin (vitamin B_2_) is a direct precursor to the cofactors flavin mononucleotide (FMN) and flavin adenine dinucleotide (FAD). Riboflavin is synthesized by plants and many microorganisms; it is not synthesized by animals [1]. Many Gram-positive bacteria seem to be capable of acquiring riboflavin from the environment, whereas most Gram-negative bacteria depend on the endogenous synthesis of this vitamin [2]. Riboflavin transporters (uptake systems) have been identified and characterized in *B. subtilis *[3], in *Lactococcus lactis *[4,5] and in a few other bacteria. Three classes of riboflavin transporters seem to exist: (1) homologs of *ribU *of *B. subtilis*, (2) homologs of *ribM *of *Corynebacterium glutamicum *and (3) homologs of *impX *of *Fusobacterium nucleatum *[6]. The latter class has not been functionally characterized [2]. *B. subtilis *RibU is part of a modular multi-subunit riboflavin transporter and belongs to the recently identified family of energy-coupling factor (ECF) transporters [7-11]. *L. lactis *RibU [4] has also been included in the latter classification, the driving force behind transport activity was shown to be ATP hydrolysis [9]. Notably, RibU of *Staphylococcus aureus *has been crystallized and its three-dimensional structure has been determined [8]. *B. subtilis *RibU is a proton-riboflavin symporter with high affinity for its substrate (*K_m _*= 5-20 nM) [3]. RibU is strikingly different from the *Corynebacterium glutamicum *riboflavin transporter RibM, which was characterized as an energy-independent facilitator for riboflavin with much lower affinity (*K_m _*= 11 μM) [3]. RibM from *C. glutamicum *is similar (40% at the amino acid level) to RibM (23.7 kDa) from *S. davawensis*. The gene for the latter protein is present in the *S. davawensis *riboflavin biosynthetic gene cluster *ribBMAH *which is controlled by an FMN riboswitch [12] directly upstream of *ribB *[13]. *S. davawensis *is the only known producer of the riboflavin analog roseoflavin, which has antibiotic activity [14]. Highly similar (> 65% similarity) RibM proteins (all containing five putative trans membrane domains) are present in other species of the genus *Streptomyces*. The gene for the flavin facilitator *ribM *from *S. davawensis *was codon optimized for expression in *B. subtilis*. The gene *ribM *was functionally characterized and was found to encode a transporter for riboflavin and roseoflavin. Finally, *ribM *was evaluated as a possible tool to enhance the productivity of a *B. subtilis *riboflavin production strain.

## Results

### Expression of *ribM *from *S. davawensis *in *E. coli *increased riboflavin uptake

**Figure 1:**
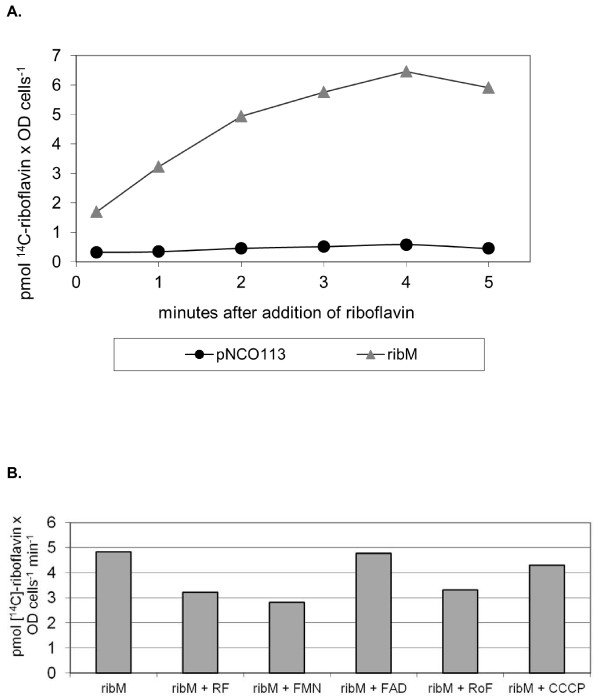
**Characterization of RibM from *Streptomyces davawensis *as a riboflavin transporter**. (A) The gene *ribM *from *S. davawensis *was overexpressed in *E. coli *BL21 using the plasmid pNCO113ribM (grey triangles). An *Escherichia coli *strain containing the empty vector pNCO113 (black circles) served as a control. *E. coli *strains were grown in riboflavin-free M9 medium. The cells were collected by centrifugation, suspended in transport buffer (50 mM K_2_HPO_4_/KH_2_PO_4_, 50 mM MgCl_2_, pH 7.0) and the experiment was started by adding [^14^C]riboflavin to a final concentration of 1.6 μM. Samples were filtered, washed with water and the radioactivity was determined by liquid scintillation counting. "1 OD cells" means "the amount of cells that when present in 1 ml will lead to an OD_600 _reading of 1.0". (B) Uptake experiments were performed as described for (A) with 2.2 μM [^14^C]riboflavin in the presence of a 10-fold excess of riboflavin (ribM+RF), flavin mononucleotide (ribM+FMN), flavin adenine dinucleotide (ribM+FAD), roseoflavin (ribM+RoF) or carbonyl cyanide *m*-chlorophenylhydrazone (ribM+CCCP; 130 μM). The uptake activity in the absence of competitors was 4.8 pmol pmol riboflavin × OD cells^-1 ^min^-1 ^(ribM). In the presence of CCCP uptake activity was 4.3 pmol riboflavin × OD cells^-1 ^min^-1^. "1 OD cells" means "the amount of cells that when present in 1 ml will lead to an OD_600 _reading of 1.0".

The wild-type gene *ribM *from *S. davawensis *was expressed in *E. coli *using the plasmid pNCO113ribM, employing an IPTG controlled T5 promotor [15]. *E. coli *does not contain an endogenous uptake system for flavins [16]. Riboflavin import was monitored using [^14^C]riboflavin. It was found that expression of *ribM *resulted in a significant increase in the cellular radioactivity shortly after adding the labelled substrate (Figure 1A) strongly suggesting that *ribM *codes for a functional flavin transporter. The uptake activity was 1.5 pmol riboflavin × OD cells^-1 ^min^-1 ^in the *ribM *expressing strain. The control strain (not containing *ribM*) showed an uptake activity of 0.04 pmol riboflavin × OD cells^-1 ^min^-1^. Riboflavin uptake mediated by *S. davawensis *RibM was only slightly affected by FAD and the protonophore CCCP (Figure 1B). Roseoflavin, FMN and a 10-fold excess of unlabelled riboflavin apparently reduced [14C]riboflavin uptake suggesting that roseoflavin and FMN are substrates for RibM. Similar results were generated in our previous study analysing RibM from *C. glutamicum *[3], where it was additionally shown that riboflavin uptake was not affected by the absence of glucose or the addition of sodium azide. In summary, we conclude that RibM from *S. davawensis *is a transporter for riboflavin and roseoflavin, and that transport appears to occur independently of metabolic energy.

### Introduction of *ribM *from *S. davawensis *in *B. subtilis*

The *ribM *gene from *S. davawensis *has a relatively high G+C-content (72%) and was optimized with respect to the codon usage of *B. subtilis *in order to allow efficient heterologous expression. The artificial gene was named *ribM_opt _*and was inserted into the expression vector pHT01 to give pHT01ribM_opt_. Expression using pHT01 is based on the strong σ^A^-dependent promoter (P*_grac_*) preceding the *groE *operon of *B. subtilis *which has been converted into an efficiently controllable (IPTG-inducible) promoter by addition of the *lac *operator from *E. coli*. A *B. subtilis *wild-type strain (Marburg 168) and a riboflavin overproducing *B. subtilis *strain (BSHP) were transformed with pHT01ribM_opt_. From the resulting strains, *B. subtilis *168 < pHT01ribM_opt _> and BSHP < pHT01ribM_opt _> plasmids were isolated. DNA sequencing revealed the presence of the *ribM_opt _*gene under control of P*_grac _*in all strains.

### The gene *ribM_opt _*from *S. davawensis *was functionally expressed in *B. subtilis*

**Figure 2:**
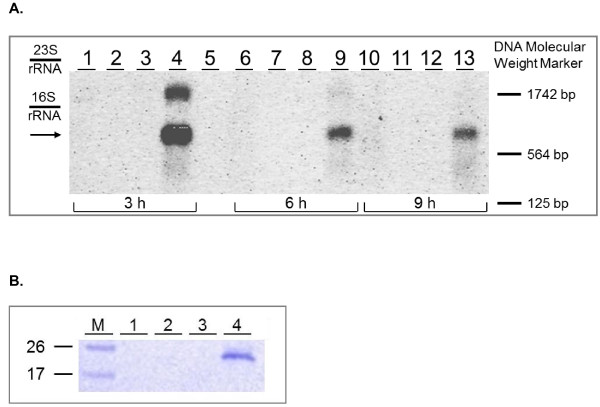
**Monitoring expression of the gene *ribM *from *Streptomyces davawensis *in recombinant *Bacillus subtilis *strains containing the plasmid pHT01ribM_opt_**. (A) Cell samples (normalized to an OD_600 _of 1) were taken 3, 6 and 9 h after treatment with IPTG and total RNA was prepared. Control cells were not treated with the inducer. The level of *ribM_opt_*-transcript was assessed by Northern blot analysis using a *ribM_opt_*-specific digoxigenin labelled DNA probe. The predicted size of the *ribM_opt _*transcript is 0.8 kb (see arrow). Lanes 1, 6 and 10: *B. subtilis *168 < pHT01 >; Lanes 2, 7 and 11: *B. subtilis *168 < pHT01 > induced with 1 mM IPTG; Lanes 3, 8, and 12: *B. subtilis *168 < pHT01ribM_opt _>; Lanes 4, 9 and 13: *B. subtilis *168 < pHT01ribM_opt _> induced with 1 mM IPTG; Lane 5, no sample. (B) After growth in LB and treatment with IPTG *B. subtilis *< pHT01ribM_opt _> cells were collected and cell free extracts were prepared. Cytoplasmic fractions and membrane fractions were subjected to SDS-PAGE, followed by transfer to a nitrocellulose membrane and analysis with anti-penta-his mouse monoclonal antibodies/goat anti-mouse IgG alkaline phosphatase (AP)-coupled secondary antibodies. Lane M, protein marker (in kDa); lane 1, *B. subtilis *< pHT01 > cytoplasmic fraction; lane 2, *B. subtilis *168 < pHT01 > membrane fraction; lane 3, *B. subtilis *168 < pHT01ribM_opt _> cytoplasmic fraction; lane 4, *B. subtilis *168 < pHT01ribM_opt _> membrane fraction; (C-terminally his_6_-tagged RibM from *Streptomyces davawensis*, calculated molecular mass: 24.7 kDa).

*B. subtilis *168 < pHT01ribM_opt _> and *B. subtilis *168 < pHT01 > (control) were grown to the exponential growth phase in LB and treated with IPTG (100 μM). Cells were harvested at different time points after induction (3 h, 6 h and 9 h) and total RNA was prepared. Northern blot analysis using a *ribM_opt _*specific oligonucleotide probe revealed, that the heterologous gene *ribM_opt _*was expressed in *B. subtilis *168 < pHT01ribM_opt _> (Figure 2A). The 0.8 kb transcript was found in IPTG treated cells only. The strongest signal was observed 3 h after induction (Figure 2A, lane 4). In this lane an additional band at 1.8 kb appeared, possibly formed by aggregates of not fully denatured *ribM_opt _*transcripts. The data showed that the *ribM_opt _*transcript was present even 9 h after induction (in the stationary growth phase). Subsequently, cytoplasmic fractions and membrane fractions of cell-free extracts of *B. subtilis *168 < pHT01ribM_opt _> and *B. subtilis *168 < pHT01 > (control) were subjected to Western blot analysis. The synthesis of his_6_-tagged RibM was monitored using anti-penta-his antibodies (Figure 2B). His_6_-tagged RibM was found in the membrane fraction of IPTG (100 μM) treated *B. subtilis *168 < pHT01ribM_opt _> only, indicating that RibM was directed to the membranes (Figure 2B, lane 4). The addition of higher amounts of IPTG (1 mM) did not enhance the amount of heterologous RibM in recombinant *B. subtilis *strains (data not shown). Thus (if not otherwise indicated), in the following experiments, stimulation of RibM synthesis in *B. subtilis *routinely was carried out using 100 μM IPTG.

### Synthesis of RibM in *B. subtilis *mutants supported growth and enhanced roseoflavin sensitivity

**Figure 3:**
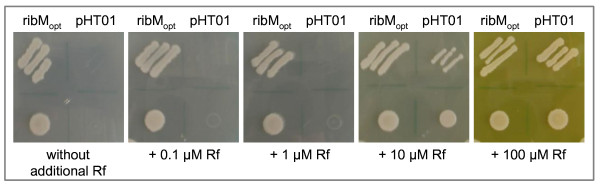
**The overproduction of RibM allowed growth of a Δ*ribU*::Kan^r ^Δ*ribB*::Erm^r ^*Bacillus subtilis *strain**. Streaks (top) and drops (bottom, about 50,000 cells) of *B. subtilis *Δ*ribU*::Kan^r ^Δ*ribB*::Erm^r ^cells expressing *ribM *from plasmid pHT01ribM_opt _were applied to LB plates (about 0.5 μM riboflavin) containing the indicated (additional) amount of riboflavin and 100 μM IPTG. Growth was recorded after incubation for 36 h at 37°C. As controls, strains were transformed with the empty vector pHT01. Apparently, only the strains transformed with pHT01ribM_opt _could grow on culture media (LB) with low amounts of riboflavin.

In the following experiment the *B. subtilis *double mutant Δ*ribB*::Erm^r ^(*ribB *was deleted and instead an erythromycin resistance gene was introduced) Δ*ribU*::Kan^r ^(*ribU *was deleted and replaced by a kanamycin resistance gene) was used. This strain carries deletions in *ribB*, encoding riboflavin synthase (EC 2.5.1.9) catalyzing the last step of riboflavin biosynthesis [17], and in *ribU*, the energy-dependent riboflavin transporter [3]. The double mutant was transformed with pHT01ribM_opt _or the empty plasmid and both transformants were compared in growth assays on LB plates. Only the strains transformed with pHT01ribM_opt _could grow on LB, which contains about 0.5 μM riboflavin, or on LB supplemented with (additional) 0.1 μM riboflavin or 1 μM riboflavin (Figure 3). Upon addition of 10 μM riboflavin both transformants could grow. Growth of the control strain, however, was reduced. At 100 μM riboflavin no difference in growth was observed indicating that at this concentration the vitamin crosses the cytoplasmic membrane independently of a dedicated transport system. Similar experiments were performed in liquid Spizizen minimal media containing glucose or sucrose (both at 10 g/L) and 1 μM riboflavin in the presence or absence of the inducer IPTG. In all experiments, only the pHT01ribM_opt _containing strains were able to grow (data not shown). We conclude from these experiments that RibM was responsible for flavin transport, giving further evidence that RibM from *S. davawensis *is a functional transporter in *B. subtilis*.

**Figure 4:**
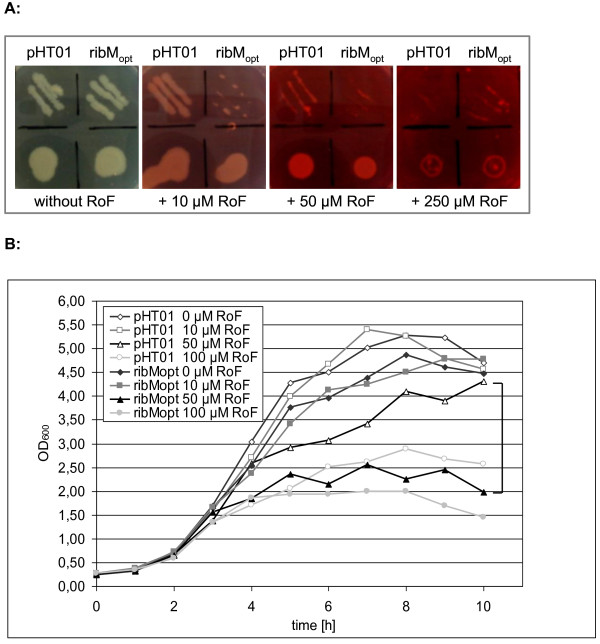
**Overproduction of RibM enhanced roseoflavin sensitivity of a Δ*ribU*::Kan^r ^*B. subtilis *strain**. (A) Streaks (top) and drops (bottom, about 50,000 cells) of *B. subtilis ΔribU*::Kan^R ^cells expressing *ribM *from plasmid pHT01ribM_opt _were applied to LB plates containing the indicated amounts of the toxic riboflavin analog roseoflavin and 100 μM IPTG. Growth was recorded after incubation for 36 h at 37°C. As controls, strains were transformed with the empty vector pHT01. At 10 μM roseoflavin it was most obvious that the presence of RibM increased roseoflavin sensitivity due to import of the toxic compound. (B) *B. subtilis ΔribU::Kan^R ^*expressing *ribM *from plasmid pHT01ribM_opt _was grown in LB broth in the presence of the indicated amounts of roseoflavin and 100 μM IPTG. Growth was recorded at μ = 600 nm. As controls, *ΔribU::Kan^R ^*strains containing empty pHT01 were analyzed. At 50 μM roseoflavin, the effect of RibM was most obvious (see bracket). Cells (black triangles) producing RibM imported toxic roseoflavin and consequently grew to an OD_600 _of about 2.5 only. The control strain (open triangles) was less affected by roseoflavin and grew to an OD_600 _of 4.2.

The mutant strain Δ*ribU*::Kan^r ^(deficient in flavin uptake) was transformed with pHT01ribM_opt _and tested with respect to roseoflavin sensitivity on LB plates containing 100 μM IPTG (Figure 4A). Roseoflavin is a toxic riboflavin analog. Clearly, the presence of *ribM *enhanced roseoflavin transport and thus roseoflavin sensitivity of Δ*ribU*::Kan^r ^< pHT01ribM_opt _> at 10 μM roseoflavin. The addition of > 50 μM roseoflavin reduced growth of the Δ*ribU*::Kan^r ^control strain containing empty pHT01, indicating that the flavin (like riboflavin) is able to permeate over the cytoplasmic membrane without catalysis mediated by a facilitator. Similar experiments were carried out using liquid LB cultures. *B. subtilis *Δ*ribU*::Kan^r ^containing pHT01ribM_opt _was grown in the presence of the inducer IPTG and different concentrations of roseoflavin (Figure 4B). In LB broth, the effect of *ribM_opt _*was most obvious at 50 μM roseoflavin where the *ribM_opt _*containing strain grew to OD_600 _of about 2.5 and the strain containing the empty vector grew to OD_600 _of 4.2. A higher concentration of roseoflavin (100 μM) inhibited growth of both strains, pointing towards a transporter independent diffusion of roseoflavin (as observed for riboflavin).

### RibM enhanced riboflavin production in a high performance *B. subtilis *production strain

**Figure 5:**
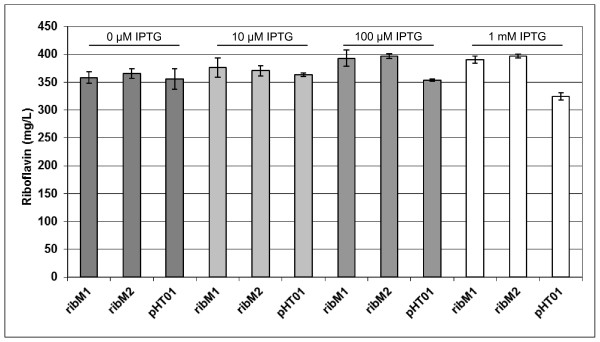
**Introduction of the heterologous flavin facilitator RibM from *Streptomyces davawensis *enhanced riboflavin synthesis in the high-performance riboflavin production strain BSHP**. Two BSHP strains containing pHT01ribM_opt _(designated as ribM1 and ribM2) and one strain containing pHT01 (control) were grown in shake flasks in the medium used for industrial riboflavin production. For each strain three shake flask experiments were carried out (n = 3). Different amounts of IPTG were used to induce synthesis of RibM. At the end of fermentation (30 h) residual sugar and the optical density at μ = 600 nm were measured. In all shake flasks the carbon source was completely metabolized and the strains had grown to a very similar cell density. For the cultures induced with 100 μM and 1 mM IPTG the t test gave the result that the RibM containing strains produced more riboflavin (mg/L), which was significant at the 5% level.

The high-performance riboflavin production strain BSHP, which produces about 350 mg/L riboflavin, was transformed with pHT01ribM_opt _and pHT01. Two transformant BSHP strains containing pHT01ribM_opt _("ribM1" and "ribM2") and four transformant strains containing pHT01 (controls) were isolated and grown in shake flasks in a medium routinely used for the analysis of the performance of industrial riboflavin production strains. For each strain three different flasks were inoculated and analyzed (n = 3). The cells were treated with IPTG in order to control synthesis of RibM. The final cell densities of all cultures were very similar (about OD_600 _of 4). At the end of fermentation residual sugar was determined. In all cultures the carbon source was completely metabolized. The total amount of riboflavin (mg/L) (intracellular riboflavin and riboflavin present in the fermentation broth) in the cultures was measured (Figure 5). At 0 μM IPTG the riboflavin concentration was similar in cultures of the pHT01ribM_opt _containing strains ribM1 and ribM2 when compared to the control pHT01 (the results of the three other control strains were very similar and thus the data were not included in the figure). Similar results were obtained at 10 μM IPTG. At 100 μM IPTG and at 1 mM IPTG, however, a significant increase at the 5% level of significance was found. The data revealed that the differences in productivity were 11% (100 μM IPTG) and 18% (1 mM IPTG). The latter values were determined by comparing the average amount of riboflavin synthesized by ribM1 and ribM2 to the amount produced by the control. Surprisingly, at 1 mM IPTG the amount of riboflavin in cultures of the control strain containing the empty plasmid pHT01 was significantly lower as compared to the values obtained at 0 μM IPTG, 10 μM IPTG and 100 μM IPTG. We do not have an explanation for this. As mentioned above, the control experiment was done with four different independent transformant strains with similar results. It is known that reduced growth positively affects the accumulation of metabolites. Growth, however, was very similar in all cultures (the carbon source was completely metabolized) and therefore a growth limitation cannot explain the apparent increase of the riboflavin synthesis. All in all our data suggest that the presence of RibM enhanced riboflavin production under these experimental conditions.

## Discussion

Our results suggest that RibM from *S. davawensis *mediates flavin (riboflavin/roseoflavin) translocation *via *an energy independent facilitated diffusion mechanism. The probable physiological role of RibM is the acquirement of riboflavin from the environment. The genes *ribBMAH *from *S. davawensis *form a transcription unit and are expressed only when riboflavin is limiting in the growth medium [13]. The genes *ribB *(riboflavin synthase, alpha-chain; EC 2.5.1.9), *ribA *(bifunctional GTP cyclohydrolase II/3,4-dihydroxy-2-butanone-4-phosphate synthase; EC 3.5.4.25) and *ribH *(lumazine synthase; EC 2.5.1.9) are responsible for riboflavin biosynthesis. The fact that *ribM *is cotranscribed with the genes *ribB*, *ribA *and *ribH *suggests that a transporter is produced in parallel to the riboflavin biosynthetic enzymes. This appears not to be economical. If no riboflavin is present in the cytoplasm, the corresponding biosynthetic enzymes must be synthesized. The coproduction of a riboflavin transporter, however, is reasonable only when external riboflavin indeed is present. In turn, if riboflavin is present in the growth medium, the production of biosynthetic enzymes apparently constitutes a waste of metabolic energy. Notably, in the Streptomycetes *S. avermitilis*, *S. coelicolor*, *S. scabiei *and *S. griseus ribBMAH *clusters (containing *ribM *transporter genes) are present as well. It seems that Streptomycetes play it safe with respect to riboflavin supply. If riboflavin is growth limiting, proteins for transport and biosynthesis are synthesized in parallel to ensure availability of the vitamin.

The transport activity and direction of an energy independent facilitator depends on the concentration of the metabolite in the cytoplasm and the surrounding medium, respectively. Thus, RibM in principle may also confer roseoflavin resistance to *S. davawensis *(by exporting roseoflavin from the cytoplasm), which naturally produces this antibiotic in the stationary growth phase. The export activity of RibM (or other riboflavin transporters) in principle may also be of importance with respect to metabolic engineering of riboflavin production strains. In order to test this idea, *ribM *was functionally expressed in a production strain of *B. subtilis*, an organism, which is used for the commercial production of riboflavin [18]. The introduction of *ribM *into a high performance *B. subtilis *riboflavin production strain apparently improved riboflavin production. Although the increase was relatively small, for a large scale production process the improvement may be relevant. We suggest that the export activity of RibM leads to reduced levels of riboflavin in the cytoplasm possibly enhancing the carbon flux through the pathway. This may enhance productivity during the exponential growth phase (when riboflavin concentration outside is low) or during stationary growth (when riboflavin levels inside the cells are extremely high). Wild-type *B. subtilis *cells rapidly and almost quantitatively convert intracellular riboflavin to FMN and FAD [19], a reaction, which is catalyzed by the bifunctional flavokinase/FAD synthetase RibC. All production strains have a strongly reduced RibC activity (1%) [20,21]. Thus, production strains contain unusually high amounts of free riboflavin, which, in principle, might reduce the activity of the riboflavin biosynthetic enzymes.

The introduction of a protein catalyzing riboflavin export may also improve current production processes employing other microorganisms such as *Ashbya gossypii *or *Candida famata *[22]. For *A. gossypii *interesting physiological studies suggest riboflavin export across the plasma membrane [23]. To our knowledge the corresponding exporter has not yet been identified.

Successful examples for strain optimization on the basis of metabolite export (L-threonine and L-lysine) have been reported for *Corynebacterium glutamicum *[24-26]. Our work is the first to use a similar approach for optimizing one of the most successful biotechnological processes, the commercial synthesis of riboflavin [2].

## Conclusions

The gene *ribM *from *S. davawensis *encodes a membrane protein which is able to catalyze the uptake of riboflavin but also of the antibiotic roseoflavin, a structural riboflavin analog. The functional expression of *ribM *in *E. coli *and *B. subtilis *apparently was possible without the cosynthesis of other protein components. Furthermore, our previous [3] and our present data data suggest that RibM proteins are energy independent flavin facilitators. Consequently, *S. davawensis *RibM could in principle also catalyze riboflavin export and be useful to increase the riboflavin yield in a riboflavin production process. Most riboflavin currently is produced using genetically engineered microorganisms, whereby *B. subtilis *is an important host. Classic random mutagenesis and methods of metabolic engineering have been used in order to optimize *B. subtilis *for the production of riboflavin. Most effort was directed at impairment of regulation of the riboflavin biosynthetic operon and amplification of the copy number of the structural genes *ribGBAH(T) *[2]. However, import of substrates and export of the product have not yet been considered as strategies for further improvement. In order to partially fill this gap, we introduced *ribM *into a high-performance *B. subtilis *riboflavin production strain. We could show that the gene was actively transcribed and that the gene product RibM was directed to the cytoplasmic membrane. Shake flask experiments routinely used to evaluate the performance of riboflavin overproducing strains suggest that upon induction with 100 μM IPTG, the amount of riboflavin at the end of growth indeed is higher as compared to the controls. Possibly, riboflavin transporters from other microorganisms may even be more useful and may show even better results.

## Methods

### Bacterial strains, plasmids and growth conditions

*E. coli *DH5α was used as a host for gene cloning experiments and was aerobically grown at 37°C on lysogeny broth (LB) [27,28]. *E. coli *BL21 [29] was used as a host for the uptake experiments with [^14^C]riboflavin. A recombinant *E. coli *BL21 strain overexpressing *ribM *from *S. davawensis *was generated by transformation using the plasmid pNCO113ribM which was described earlier [13]. The plasmid pET21 was obtained from Stratagene (Waldbronn, Germany). If not otherwise indicated *B. subtilis *was aerobically grown at 37°C in 2 × Spizizen's minimal medium [30] supplemented with 0.02% casamino acids, 2% yeast extract and 10% glucose or in LB. *B. subtilis *168 (*trpC2*) [31] is a wild-type strain with respect to riboflavin biosynthesis and uptake and was used as a control. The *B. subtilis *double mutant Δ*ribB*::Erm^r ^Δ*ribU*::Kan^r ^is auxotrophic for riboflavin and does not contain a functional riboflavin uptake system (*ribU*) [3]. It was grown in media supplemented with 20 mg/L riboflavin, 1 μg/ml erythromycin and 5 μg/ml kanamycin. *B. subtilis *168 Δ*ribU*::Kan^r ^[3] was grown in media supplemented with 5 μg/ml kanamycin. The *B. subtilis *high-performance riboflavin production strain BSHP was constructed by introducing additional copies of the *B. subtilis ribGBAHT *genes controlled by strong constitutive phage promoters (P*_spo_*) into the genome of *B. subtilis *strain 3979 [18,32].

### Expression of *S. davawensis ribM *in *B. subtilis*

*B. subtilis *strains overproducing RibM (*B. subtilis *< pHT01ribM_opt _>) were generated using the expression vector pHT01 (Mobitech, Göttingen, Germany) replicating in *Bacillus *species from the pUB110 origin [33]. The gene *ribM *from *S. davawensis *was codon-optimized by GENEART (Regensburg, Germany) using overlapping oligonucleotides and PCR. In addition, codons specifying a his_6_-tag were introduced at the 3'-end. The gene (*ribM_opt_*) (# FR719838, European Nucleotide Archive) was delivered in pGA4ribM_opt_. The latter plasmid was used as a template for PCR amplification of *ribM_opt _*using the modifying oligonucleotides RibMopt fw BamHI (5'-ACA GGA TCC ATG AAT TGG CTG AAT AGC-3') and RibMopt rv AatII (5'-ATT GAC GTC CTA TTA GTG GTG GTG ATG GTG-3'). The PCR product was purified and digested with *Bam*HI and *Aat*II to allow cloning in pHT01. The resulting plasmid pHT01ribM_opt _was used to transform *B. subtilis *using a standard protocol [34]. Expression of *ribM_opt _*in *B. subtilis *was stimulated by adding IPTG in the early exponential growth phase. For selection of pHT01ribM_opt _30 μg ml^-1 ^chloramphenicol was added to the growth media. Growth was monitored using a photometer at μ = 600 nm.

### Uptake experiments in *E. coli*

*E. coli *BL21 cells were grown in M9 minimal medium [27] supplemented with antibiotics as required. The growth medium was inoculated to an OD_600 _of 0.15, and the cells were grown at 37°C until they reached an OD_600 _of 0.5. *E. coli *cells were treated with 0.5 mM IPTG and grown for 3 h (to an OD_600 _of 0.8). Cells were harvested, washed once with ice-cold water and once with transport buffer (50 mM KH_2_PO_4_/K_2_HPO_4_, 50 mM MgCl_2 _pH 7.0). The cells were finally resuspended in transport buffer (10 OD_600 _ml^-1^) and stored on ice. Uptake experiments were performed with 500 μl of cells that were vigorously stirred at 30°C. After warming for 2 min, glucose was added to a final concentration of 1 mM, and the assay was started by adding [^14^C]riboflavin (specific activity, 5.54 MBq/mg; a generous gift of R. Krämer, Köln, Germany) to a final concentration of 2 μM. Several aliquots were removed at minute intervals, filtered on 0.45-μm GN-6 membrane filters (Pall, Dreieich, Germany), washed with an excess of water, and analysed by liquid scintillation counting. The transport activity was determined without further additions or after adding carbonyl cyanide *m*-chlorophenylhydrazone (CCCP; 130 μM), unlabeled riboflavin, FMN, FAD or roseoflavin (MP Biomedicals, Montreal, Canada) 3 min before addition of the labeled substrate. The latter assay was done only once.

### RNA preparation, Northern (RNA) blotting and hybridisation

The preparation of total RNA from *B. subtilis*, the transfer of RNA to a solid support and the hybridisation to a *ribM_opt _*specific digoxigenin-labelled DNA probe was carried out according to standard procedures [27]. The detection of the digoxigenin labelled probe was performed as suggested by the supplier of the "DIG DNA Labeling and Detection Kit" (Roche Diagnostics, Mannheim, Germany).

### Preparation of *B. subtilis *membranes and Western blot analysis

Stationary phase cultures (50 ml) were harvested by centrifugation and washed twice with cold 50 mM Tris-HCl (pH 8.0). Pellets were dissolved in 1 ml TMS buffer (50 mM Tris-HCl, pH 8.0; 16 mM MgCl_2_; 33% sucrose (w/v); 300 μg/ml lysozyme; 1 mM phenylmethanesulfonylfluoride, PMSF) and incubated for 60 min at 37°C. Protoplasts were harvested by centrifugation (10 min at 7,500 × g at 4°C). Pellets were suspended in 1 ml lysis buffer (50 mM Tris-HCl, pH 8.0; 5 mM MgSO_4_; 2 mM PMSF). Cell free extracts were produced by sonication (1 min at 60% of the maximal power and 50% interval) on ice. Membranes were collected by ultracentrifugation for 30 min at 100,000 × g and washed once with 50 mM Tris-HCl (pH 8.0). Pellets were dissolved in 50 mM Tris-HCl (pH 8.0). The protein concentration was estimated by the method of Bradford [35]. The samples were analyzed by SDS-PAGE on 4-20% gradient polyacrylamide gels using 50 μg of protein per lane. After transfer to nitrocellulose membranes his_6_-tagged RibM was immunologically detected (mouse anti-penta-his primary antibodies/goat anti-mouse IgG alkaline phosphatase(AP)-coupled secondary antibodies; Novagen, Darmstadt, Germany). AP was detected with the "AP Detection Reagent Kit" (Novagen) using 3-bromo-4-chloro-5-indolyl phosphate and nitro blue tetrazolium chloride.

### Monitoring of riboflavin production

Riboflavin synthesis by *B. subtilis *in the time course of fermentation was monitored as follows. An aliquot from the culture containing cells and medium (500 μl) was combined with 465 μl 4 N NaOH and vigorously mixed for 1 min. The mixture was neutralized by adding potassium phosphate (1 M pH 8.0) and centrifuged for 5 min at 13,000 × g at room temperature. Riboflavin in the supernatant was determined by using a standard procedure employing HPLC [21]. Using the above described protocol the total amount of riboflavin (intracellular riboflavin and riboflavin present in the fermentation broth) was measured. For the statistical analysis of the data student's t test was applied comparing two unknown means based on independent samples.

## Authors' contributions

SH was involved in plasmid and strain construction, performed the Northern blot experiments and generated the membrane preparation/Western blot data. DP performed the growth experiments in the presence of flavin analogs. JS and CV did the riboflavin uptake experiments in *Escherichia coli*. ML carried out the experiments with the riboflavin production strain. MM coordinated the experiments and drafted the manuscript. All authors have read and approved the final version of the manuscript.
